# Post-occlusion hyperemia ASL differentiates peripheral artery disease from controls

**DOI:** 10.1186/1532-429X-16-S1-P10

**Published:** 2014-01-16

**Authors:** David Lopez, Craig H Meyer, Frederick H Epstein, John M Christopher, Jennifer Kay, Christopher M Kramer

**Affiliations:** 1 Medicine, University of Virginia, Charlottesville, Virginia, USA; 2 Biomedical Engineering, University of Virginia, Charlottesville, Virginia, USA; 3 Radiology and Medical Imaging, University of Virginia, Charlottesville, Virginia, USA

## Background

Pulsed arterial spin labeling (PASL) is a non-contrast MRI technique that can be used to quantify calf muscle perfusion with stress. We hypothesized that peak post-occlusion hyperemia measured with PASL would discriminate peripheral artery disease (PAD) patients from healthy subjects (NL).

## Methods

Ten NL (70% M) and 11 PAD (81% M) volunteers were enrolled. Limb ischemia was induced with a thigh cuff for 5 minutes. After inflation to 200-230 mmHg, absence of residual flow was verified with axial SSFP cine images. Immediately after cuff deflation, 15 averaged PASL images were acquired using a single-shot echo-planar (EPI) pulse sequence (total scan time 62 s, FOV 200 × 200 mm, matrix 64 × 64, TR 4000 ms, TE 32 ms, 10 mm thick). PASL was performed using the PICORE technique with proximal blood labeling. The Q2TIPS technique minimized errors from variable transit delay of spins from labeling region to imaging slice and contamination of perfusion signal by intravascular blood. Flow was measured by drawing regions of interest on the motion corrected EPI while avoiding large blood vessels, and then copied to the flow maps. Flow measurements were compared for individual muscle groups and as a composite of all calf muscle groups.

## Results

**Figure 1:**
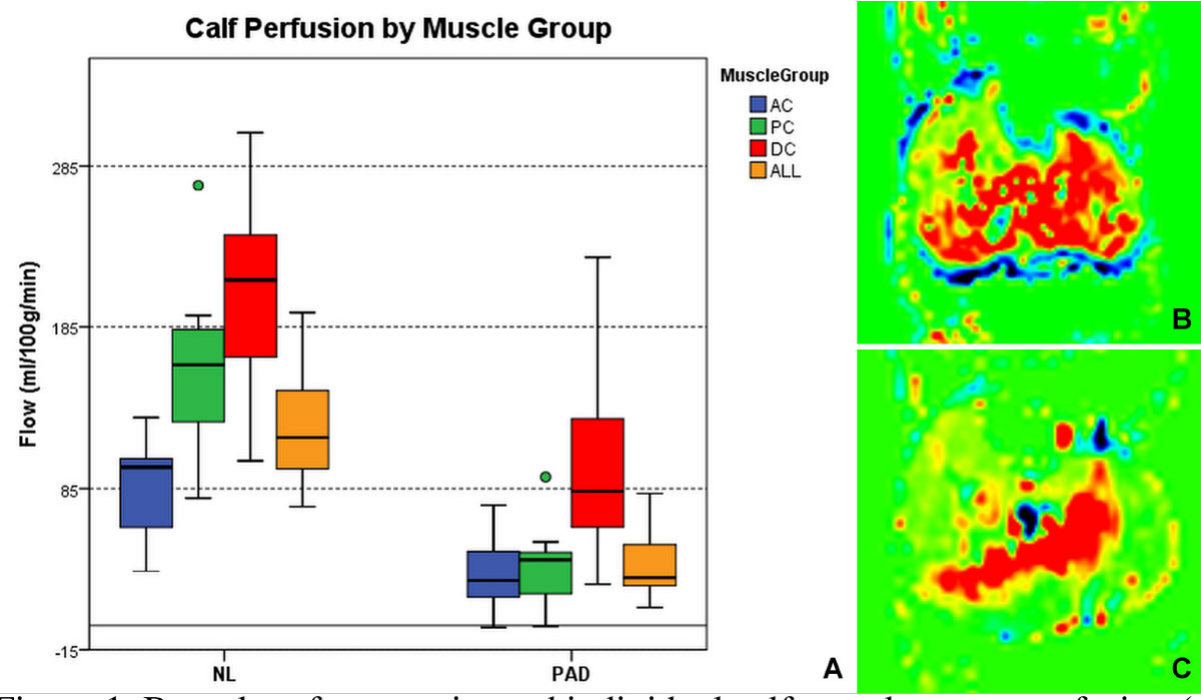
**Box plot of composite and individual calf muscle group perfusion (A)**. Note how the DC flow in PAD patients is similar to AC flow in NL. While reactive hyperemia was notable in all compartments of NL subjects (B), it was limited to the DC in PAD patients (C). AC: anterior compartment; PC: posterior compartment; DC: deep compartment; ALL: average peak flow of all calf muscle compartments; NL: healthy volunteers; PAD: peripheral arterial disease patients.

NL subjects were younger (54 ± 7 vs. 72 ± 11, p < 0.001) and had normal ankle-brachial index (1.0 ± .07 vs. 0.68 ± .05, p < 0.001) compared to PAD. Perfusion was higher in the NL group than in PAD patients when compared by individual muscle groups (Table 1) and when the composite calf perfusion was calculated (122 ± 35 vs. 36 ± 19 mL/100 g/min, p < 0.001), mean difference 86 mL/100 g/min (95% CI 60-112)(Figure 1A). Reactive hyperemia was typically seen in all muscle groups of NL subjects (Figure 1B), whereas in PAD patients the response was limited to the deep compartment (DC) (Figure 1C). Notably, the perfusion in the DC of PAD patients was not different from the anterior compartment (AC) flow in NL volunteers (96 ± 65 vs. 88 ± 31 ml/100 g/min, p = 0.78).

**Table 1:** Composite and individual muscle group perfusion.

Peak Flow (ml/100 g/min)	NL (n = 10)	PAD (n = 11)	p-value*
Composite	122 ± 35	36 ± 22	< 0.001

Anterior Compartment	88 ± 31	32 ± 25	< 0.001

Posterior Compartment	160 ± 53	35 ± 26	< 0.001

Deep Compartment	206 ± 67	95 ± 66	0.005

*Two-sided t-test.

## Conclusions

PASL clearly differentiates PAD patients from NL volunteers during post-cuff occlusion hyperemia. However, individual muscle group measurements must be compared to reference values of the same compartment since PAD patients may have normal range hyperemia in the deep compartment. PASL during cuff-occlusion hyperemia appears to be a valuable tool for testing novel PAD therapies.

## Funding

T32 EB003841 (DL), NIH HL075792 (CMK).

